# SNHG15 Mediates MTSS1 Gene Expression via Interacting with the Gene Promoter and Regulating Transcription Pausing

**DOI:** 10.3390/ijms252111565

**Published:** 2024-10-28

**Authors:** Yanchun Zhou, Shaoying Chen, Weibin Chen, Jundong Wu, Wei Gu

**Affiliations:** Key Immunopathology Laboratory of Guangdong Province, Department of Pathophysiology, Shantou University Medical College, Shantou 515041, China; zyc2013st@foxmail.com (Y.Z.); sychen3@stu.edu.cn (S.C.); wbcu@stu.edu.cn (W.C.)

**Keywords:** LncRNA SNHG15, MTSS1, transcriptional regulation

## Abstract

Metastasis suppressor 1 (MTSS1) has been reported to play important roles in suppressing cancer progression. In this study, we investigated the underlying mechanism that regulates MTSS1 expression. We showed that in breast cancer cells, lncRNA-SNHG15-induced cell invasion and proliferation was accompanied with the decreased expression of MTSS1 mRNA. Further study revealed that SNHG15 mediated MTSS1 repression through blocking its promoter activity. Mechanistically, SNHG15 complexes with DDX5 and RTF1 and interacts with the core promoter of the MTSS1 gene to interfere with RNA-Pol-II-directed transcriptional initiation. Association with DDX5 stabilizes SNHG15 while binding to RTF1 allows SNHG15 to carry RTF1 to the core promoter, where RTF1 forms a complex with PNA pl II to enhance transcriptional pausing. Our findings revealed a molecular mechanism by which SNHG15 serves as a regulator to suppresses MTSS1 transcription via interaction with the gene core promoter.

## 1. Introduction

Breast cancer is the most common and complex disease, and metastasis is a significant contributor to death in the breast cancer patients. Although the progression of metastasis is not yet completely understood, it is known that metastasis is mediated by many factors including metastatic activators and suppressors. To date, a number of metastasis suppressor genes have been identified, many of which are associated with a variety of genetic deviations and expressive alterations during the metastatic process [1,2]. Thus, investigating the underline mechanisms by which metastasis suppressor genes are regulated would improve metastatic treatment strategies.

Metastasis suppressor 1 (MTSS1), a protein composed of 755 amino acids, was initially identified in metastatic bladder carcinoma cell lines [3]. Subsequent studies revealed that MTSS1 is associated with the inhibition of metastasis in a variety of cancers including hematopoietic and breast cancers. Accumulating evidence indicated that the function of MTSS1 to repress metastasis was through acting as a regulator that interacted with multiple partners to regulate actin dynamics [4,5]. The down-regulation of MTSS1 has been widely observed in many types of tumors, in which DNA methylation has been shown to perform a significant role to modulate its expression [6].

Recently, lncRNA (long non-coding RNA) has been considered as a common regulator to mediate gene expression. LncRNAs represent a distinct group of non-coding RNAs (ncRNAs) that exceed 200 nucleotides in length and have been reported to modulate cell proliferation, invasion, and some other fundamental biological processes at the transcriptional, post-transcriptional, and epigenetic levels [7,8]. For examples, lncRNA ANRIL modulates the PRC2 recruitment to silence tumor suppressor gene INK4B [9]; MACC1-AS1 cis-regulates MACC1 transcription by interacting with the gene promoter [10]. Increasingly, studies have shown that lncRNAs can localize either in the nucleus or in the cytoplasm and that their localization is often associated with their biological functions [11,12]. Currently, lncRNAs have been well identified and classified; many of them are still being revealed and their biological functions need to be further annotated [13].

Small nucleolar RNA host gene 15 (SNHG15) was initially characterized as a short half-life lncRNA in the study of cellular stress responses [14]. Recent studies have indicated that the up-regulation of SNHG15 is associated with the occurrence and development of multiple cancers [15,16,17]. SNHG15 plays significant roles in regulating cell proliferation and invasion via different potential mechanisms [18], and the abnormal expression of SNHG15 is associated with pathological features in breast cancer patients. Thus, further studies on the molecular mechanisms by which SNHG15 mediates cancer progression can provide new insight in the clinical prognosis and treatment of cancer patients.

In this study, we identified a novel function of SNHG15 to suppress the transcription of the MTSS1 gene in breast cancer cells. We showed that substantial quantities of SNHG15 localize in the nucleus, where SNHG15 interacts with the core promoter of the MTSS1 gene. The interaction of SNHG15 with the core promoter down-regulated MTSS1 transcription and increased the occupancy of RNA polymerase II (Pol II) at the transcription start site. We further revealed that SNHG15 forms a complex with DDX5 and RTF1. Association with DDX5 stabilized SNHG15 and binding to RTF1 enhanced the ability of SNHG15 to reduce MTSS1 expression, possibly through transporting its associated RTF1 to make contact with RNA Pol II and regulate transcription pausing of the MTSS1 gene.

## 2. Results

### 2.1. LncRNA SNHG15 Represses the Expression of Tumor Suppressor Gene MTSS1

**Figure 1:**
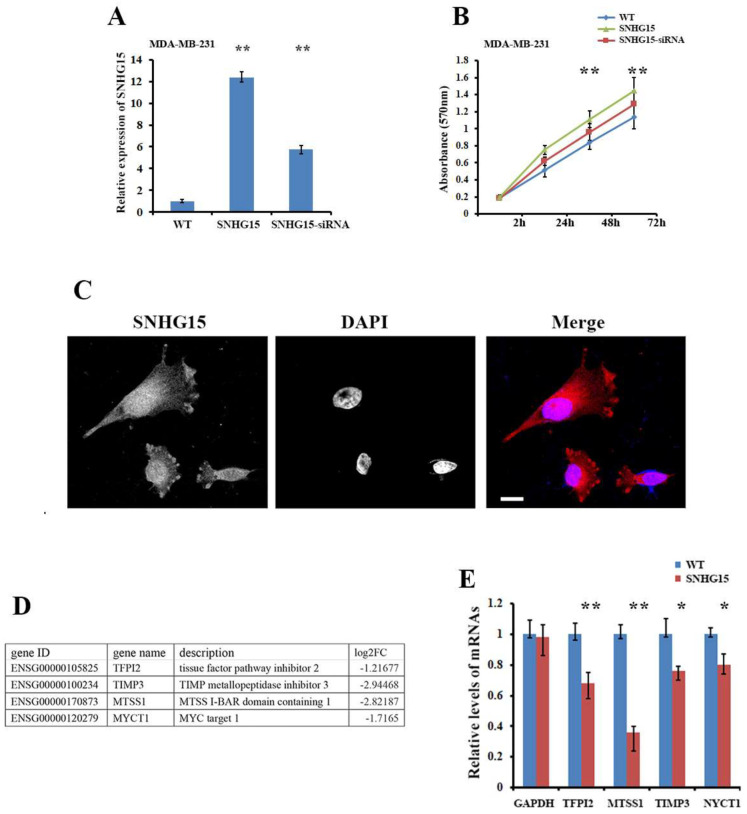
SNHG15 promotes cell proliferation and represses the expression of tumor suppressor gene MTSS1. (**A**) Expression of SNHG15 was determined by RT-qPCR in two MDA-MB-231 stable cell lines: one constantly expressed SNHG15 (MDA-MB-231/SNHG15) and the other comprised siRNA-treated MDA-MB-231/SNHG15 cells. ** *p* < 0.01. (**B**) Cell proliferation assays indicated that SNHG15 expression increased cell growth potential while knocking down SNHG15 markedly reduced proliferation ability. ** *p* < 0.01. (**C**) FISH was performed to distinguish the subcellular localization of SNHG15 in SNHG15-expressing BT549 cells. Substantial quantity of SNHG15 was localized in the nucleus. Scale bar: 10 µm. (**D**) Differential expression of genes in breast cancer cells with or without ectopic SNHG15 expression using mRNA-seq assays. A total of 99 genes were down-regulated in responding to SNHG15 expression. The table lists four down-regulated transcripts that are important for cancer progression. (**E**) Four individual transcripts and a control GAPDH mRNA were selected to confirm their expression in BT549 stable cells expressing exogenous SNHG15. qRT-PCR showed that all four selected mRNAs displayed a similar differential expression pattern as indicated in BT-549 cells. Particularly, levels of MTSS1 mRNA were largely reduced. ** *p* < 0.01, * *p* < 0.05.

SNHG15 is transcribed as an lncRNA with a length of about 983 bp [18]. The ability of SNHG15 to promote cancer cell progression was verified in breast cancer cell lines, in which the ectopic expression of SNHG15 increased cell proliferation and invasion, while these potentials could be rescued by knocking down SNHG15 (Figure 1A,B; Supplementary Figure S1A). FISH (fluorescence in situ hybridization) experiments indicated that substantial quantities of SNHG15 were localized in the nuclei of breast cancer cells expressing either ectopic SNHG15 (Figure 1C) or endogenous SNHG15 (Supplementary Figure S1B), suggesting that SNHG15 could possess a nuclear function. To investigate the molecular mechanism by which SHNG15 induces cell proliferation, we had previously examined the differential expression of genes in BT-549 cells with or without ectopic SNHG15 expression by using mRNA-seq assays (each assay was performed at the Shanghai Biotechnology Corporation, China. Data on these assays have been deposited in the Gene Expression Omnibus under submission number GSE220492; see [19]). Four mRNAs, including MTSS1 mRNA, which is important for cancer development, were down-regulated in SNHG15-expressing cells (Figure 1D). The down-regulation of these genes in responding to SNHG15 expression was further confirmed in at least two breast cancer cell lines (Figure 1E, Supplementary Figure S1C). Since the MTSS1 gene plays a vital role in breast cancer metastasis [4], we hypothesized that SNHG15-induced breast carcinogenesis could be largely contributed to by the down-regulation of MTSS1 mRNA.

### 2.2. Corelative Expression of SNHG15 and MTSS1 mRNA in Breast Cancer

**Figure 2:**
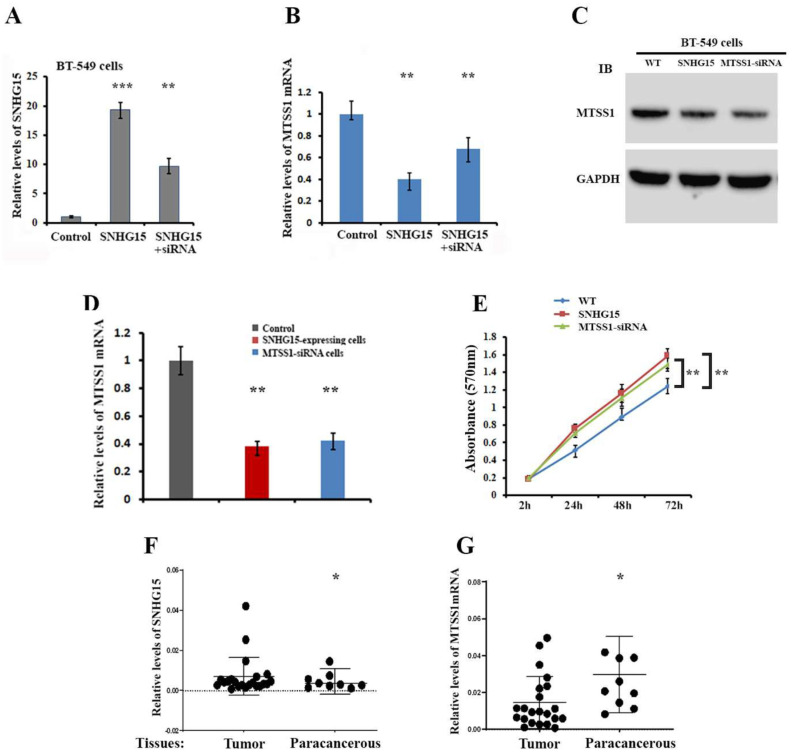
Association of SNHG15 and MTSS1 expression in breast cancer cells. (**A**) Levels of SNHG15 were determined by RT-qPCR in two BT-549 stable cells, one ectopically expressing SNHG15 and the other SNHG15-siRNA-treated. *** *p* < 0.001; ** *p* < 0.01. (**B**) qRT-PCR indicated that in responding to SNHG15 expression, MTSS1 mRNA was significantly reduced. The reduction of MTSS1 mRNA could be rescued by silencing SNHG15. ** *p* < 0.01. (**C**) Western blots showed that MTSS1 protein was markedly decreased in cells that had either over-expression of SNHG15 or silencing of MTSS1 mRNA by siRNA. (**D**,**E**) MTT assays revealed that in both SNHG15-expressing and MTSS1-silenced cells, cell proliferation was significantly increased. ** *p* < 0.01. (**F**,**G**) Total RNA was prepared from human breast tumor samples (N = 24) and paracancerous breast tissues (N = 9). Relative levels of SNHG15 and MTSS1 mRNA were measured by qRT-PCR. Raw Ct values for each gene were normalized to raw Ct values for GAPDH mRNA that was used as the internal control. Relative values are from three independent experiments. * *p* < 0.05.

Next, we used BT-549 cells to determine the expressive connection between SNHG15 and MTSS1 mRNA. RT-qPCR indicated that the cellular levels of MTSS1 mRNA were significantly reduced in cells expressing ectopic SNHG15 while the reduction could be released when SNHG15 was silenced (Figure 2A,B). As a result of SNHG15-induced MTSS1 mRNA reduction, MTSS1 protein was also lower in both SNHG15-expressing and MTSS1-siRNA treated cells (Figure 2C). Similar results were also observed in MDA-MB-231 cells where the silencing of SNHG15 by siRNA increased the levels of MTSS1 mRNA (Supplementary Figure S2). Moreover, either the over-expression of SNHG15 or the silencing of MTSS1 mRNA increased the potential of cell proliferation (Figure 2D,E), suggesting that SNHG15-mediated cell proliferation could occur mainly through the down-regulation of MTSS1 expression. We then collected some discarded human breast tumor samples and normal paracancerous tissues and utilized a qRT-PCR approach to examine the co-relative expression of MTSS1 mRNA and SNHG15. The results showed that the levels of SNHG15 in breast tumor tissues were relatively higher than in paracancerous tissues while the levels of MTSS1 mRNA were obviously lower in malignant breast tumors (Figure 2F,G), indicating a possibility that higher SNHG15 or lower MTSS1 levels could be associated with breast tumors.

### 2.3. Isolation of SNHG15 Complex Revealed That DDX5 and RTF1 Are Two Proteins Associated with SNHG15

**Figure 3:**
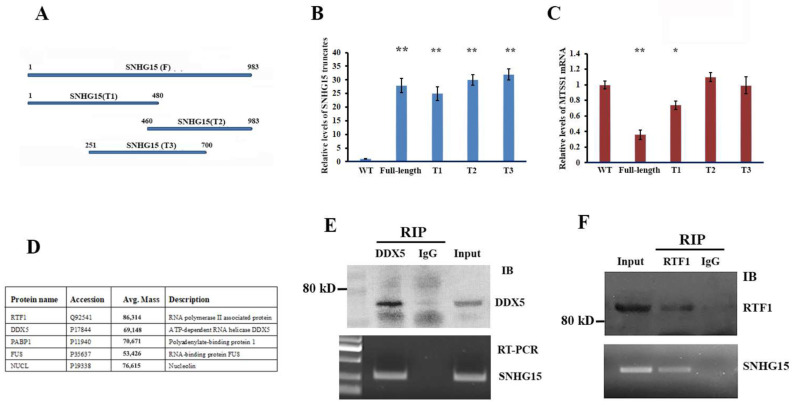
DDX5 and RTF1 are two proteins associated with SNHG15. (**A**) DNA plasmids expressing full-length SNHG15 and three truncated SNHG15 samples were constructed and stably transfected into BT-549 cells. The relative position of the truncated SNHG15 is shown. (**B**,**C**) qRT-PCR was performed to measure the relative levels of full and truncated SNHG15 and corresponding expression of MTSS1 mRNA in individual stable cells. ** *p* < 0.01. * *p* < 0.05. (**D**) SNHG15 pulldown and MS spectrometry assays were performed to identify proteins associated with SNHG15. The table lists five proteins identified to co-precipitate with SNHG15 that contain DDX5 and RTF1. (**E**,**F**) DDX5 and RTF1 antibodies were used to immune-precipitate their associated RNA targets in the nuclear extracts of breast cancer cells. Normal IgG was utilized in negative controls. SNHG15 was co-precipitated with DDX5 or RTF1.

To reveal the molecular mechanism by which SNHG15 mediates MTSS1 gene expression, we initially asked which region of SNHG15 is responsible for mediating MTSS1 mRNA expression. We dissected the SNHG15 into three truncated segments with the sizes of 1–480 bp (T1, 5′ region), 460–983 bp (T2, 3′ region) and 251–700 bp (T3, middle region) (Figure 3A). After establishing BT-549 cell lines stably expressing individual SNHG15 truncates (Figure 3B), we performed qRT-PCR tests to examine the effect of SNHG15 truncates on MTSS1 mRNA expression. In comparison to the full-length SNHG15, the expression of the T1 truncate partially decreased the levels of MTSS1 mRNA while both T2 and T3 truncates had little effect on MTSS1 mRNA expression (Figure 3C). These results indicated that the full length of SNHG15 would be required for maximally repressing MTSS1 transcription while the 5′ region also contributed some inhibitive ability for MTSS1 mRNA expression. Since ectopically expressed SNHG15 in BT549 stable cells was tagged with six MS2 hairpin repeats at the 3′ end, we next used a recombinant MBP–MCP to pull down SNHG15 (Supplementary Figure S3A). This approach would allow us to isolate SNHG15 complexes and analyze the proteins associated with SNHG15. Mass spectrometry analysis indicated that SNHG15 associated with a group of protein partners (data on the protein spectrometry were submitted to the ProtemeXchange database with the submission number IPX0005630000; see [19]). A number of proteins with higher affinity to SNHG15 were shown in Figure 3D. Two of these proteins attracted our attention: one was DDX5, an RNA-binding protein involved in many aspects of gene regulation, and another was RTF1, a protein involved in the negative regulation of RNA-polymerase-II-directed transcriptional initiation [20]. The in vivo binding of SNHG15 to DDX5 or RTF1 was further confirmed by DDX5-RIP, RTF1-RIP (Figure 3E,F), and SHNG15 pulldown assays (Supplementary Figure S3B), suggesting that DDX5 and RTF1 would play roles in SNHG15 function through forming complexes with SNHG15.

### 2.4. Roles of DDX5 and RTF1 on SNHG15-Meidated Repression of MTSS1 Expression

**Figure 4:**
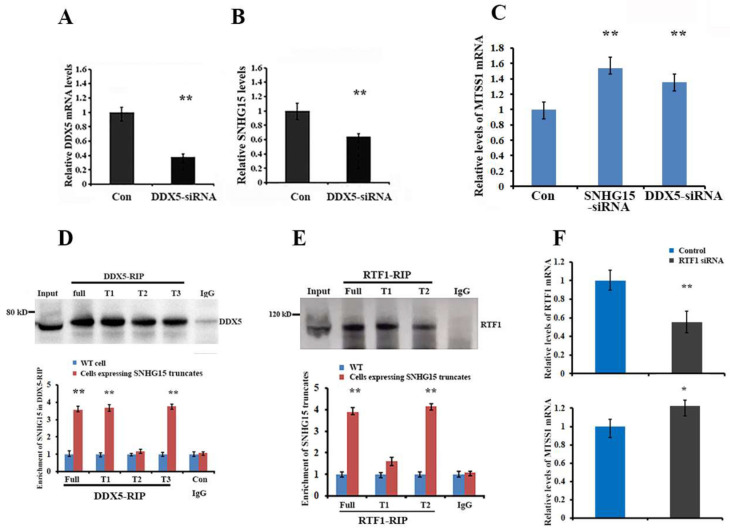
Roles of DDX5 and RTF1 in SNHG15-mediated MTSS1 mRNA expression. (**A**) qRT-PCR indicated that DDX5 mRNA was silenced by siRNA treatment. ** *p* < 0.01. (**B**) Levels of SNHG15 were decreased after knocking down of DDX5 mRNA. ** *p* < 0.01. (**C**) The role of DDX5/SNHG15 in MTSS1 mRNA expression was determined in SNHG15-expressing BT549 cells. Knocking down either SNHG15 or DDX5 increased expression of MTSS1 mRNA. ** *p* < 0.01. (**D**) DDX5-RIP was performed to detect which region of SNHG15 was binding to DDX5. The results showed that the T1 truncate or 5′ region of SNHG15 was associated with DDX5. ** *p* < 0.01. (**E**) RTF1-RIP was performed to detect the region of SNHG15 that bound to RTF1. The results showed that the T2 truncate or 3′ region of SNHG15 bound to DDX5. ** *p* < 0.01. (**F**) Effect of RTF1 on MTSS1 mRNA expression was determined by silencing of RTF1 with siRNA (upper). Knocking down of RTF1 moderately increased MTSS1 levels (lower). * *p* < 0.05, ** *p* < 0.01.

RNA-binding proteins often play important roles in the activity of their binding partners. We next determined the potential functions of DDX5 and RTF1 in SNHG15 expression. We first silenced DDX5 mRNA and performed qRT-PCR tests in breast cancer cells, which showed that the down-regulation of DDX5 reduced levels of SNHG15 (Figure 4A,B). Moreover, knocking down either SNHG15 or DDX5 mRNA increased MTSS1 expression (Figure 4C). These results suggested that the binding of DDX5 stabilized SNHG15 and maintained the function of SNHG15 to repress MTSS1 expression. To study which region of SNHG15 binds to DDX5, we performed DDX5-RIP in cells expressing individual SNHG15 truncates (refer to Figure 3A). The results showed that except the T2 truncate, the full-length, T1, and T3 truncates were enriched in the DDX5-RIP precipitates (Figure 4D), indicating that the 5′ region of SNHG15 was recognized by DDX5. RTF1-RIP indicated that RTF1 was not associated with the 5′ region of SNHG15, indicating that the 3′ region of SNHG15 was responding to interact with RTF1 (Figure 4E). By analyzing the role of RTF1 in SNHG15 function, we observed that knocking down RTF1 moderately increased SNHG15-induced MTSS1 expression (Figure 4F). Since RTF1 is a transcription-regulatory protein that interacts with RNA Pol II and regulates the pausing of RNA Pol II [20], we assumed that the association of RTF with SNHG15 is involved in the transcription repression of the MTSS1 gene.

### 2.5. SNHG15-Mediated MTSS1 Gene Repression Could Be Through Stalling RNA Pol II at the Core Promoter

**Figure 5:**
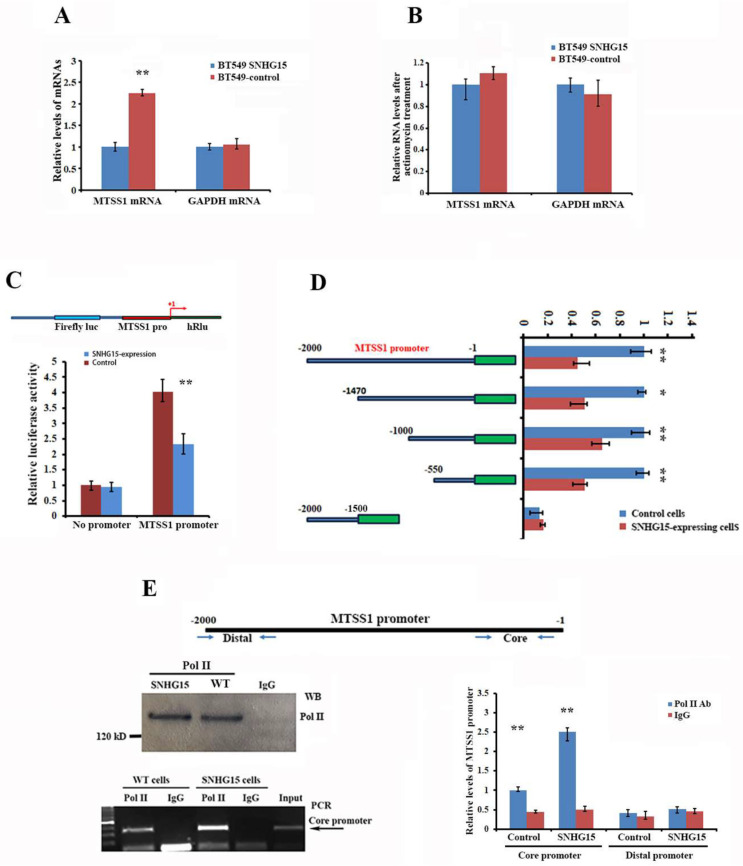
SNHG15 mediates MTSS1 gene transcription through pausing RNA Pol II at the core promoter. (**A**,**B**) Stability of MTSS1 mRNA in BT549 and BT549/SNHG15 cells was measured after treatment with actinomycin D for 16 h. Relative MTSS1 levels were determined by normalizing to GAPDH mRNA levels. Data are presented as means ± SD from three independent experiments. ** *p* < 0.01 as determined by Student’s *t*-test. ** *p* < 0.01. (**C**) Upper: a schematic luciferase reporter in which the 2000 bp of the MTSS1 promoter (MTSS1 pro) was fused into the 5′ region of the Renilla luciferase gene of the psiCHECK-2 construct. Lower: BT-549 cells with or without SNHG15 expression were transfected with the luciferase reporters. After 36 h transfection, luciferase activities were measured. Activity of renilla luciferase was normalized to firefly luciferase activity. ** *p* < 0.01. (**D**) Left: a schematic representation of luciferase reporters driven by different regions of the MTSS1-p2000 promoter. A distal region of the promoter was used as a negative control. Right: relative luciferase activity was determined in BT-549and SNHG15-expressing BT-549 cells after transfecting with the individual reporters. * *p* < 0.05, ** *p* < 0.01. (**E**) Protein A beads conjugated with RNA polymerase II antibodies were used for ChIP assays in breast cancer cells with or without SNHG15 expression. Upper penal: a schematic MTSS1 promoter (−2000 bp) in which the locations of the two set of primers for amplifying the control distal region and the core region of the MTSS1 promoter are indicated with arrows. Lower left: immunoprecipitated RNA Pol II was identified by Western blots and co-precipitated promoter regions were shown by agarose gel electrophoresis. Normal IgG was used as a negative control. Lower right: qPCR confirmed that the core promoter was preferentially co-precipitated with RNA Pol II in SNHG15-expressing cells, ** *p* < 0.01.

To eliminate the possibility that SNHG15 could post-transcriptionally regulate MTSS1 mRNA expression, we treated BT-549-WT- and SNHG15-expressing BT-549 cells with actinomycin D (5 μg/mL) for 16 h to block de novo transcription and subsequently analyzed levels of MTSS1 mRNA by RT-qPCR. Levels of MTSS1 mRNA were not significantly changed after actinomycin treatment (Figure 5A,B), indicating that SNHG15 transcriptionally mediates MTSS1 expression. To determine whether SNHG15-induced MTSS1 down-regulation was through repressing its promoter activity, we constructed a psiCHECK-2 luciferase reporter driven by a region spanning the nucleotides −2000 to −1 upstream of the MTSS1 gene (MTSS1-p2000) (Figure 5C, upper). The promoter activity was notably lower in SNHG15-expressing cells after transfection of the MTSS1-p2000 reporter into BT-549 cells for 36 h (Figure 5C). We next made a series of luciferase reporter constructs driven by different truncated MTSS1 promoters ranging from 1470 nt to −1 (MTSS1p1470), −1000 nt to −1 (MTSS1p1000), −550 nt to −1 (MTSS1p550), and −2000 to −1500 nt (MTSS1p1500) (Figure 5D, left). We presumed that the proximal −550 to −1 nt could be the core promoter because it contains a typical TAATTT box for RNA-polymerase-II binding (Supplementary Figure S4). All individual reporters containing the core promoter showed relatively lower luciferase activity in the cells expressing SNHG15. In addition, luciferase activity was greatly decreased when the core proximal promoter region was absent (Figure 5D, right). These results indicated that the core promoter region could be the regulatory region mediated by SNHG15. Since the transcription of mRNAs is generally regulated by RNA polymerase II (Pol II), which directs the initiation and elongation of gene transcription, we hypothesized that the SNHG15-mediated suppression of MTSS1 transcription could be through the way of affecting RNA Pol II function. To address this, we performed ChIP assays to analyze whether SNHG15 interferes with the binding occupancy of RNA Pol II to the core promoter. This assay was previously used to determine the pausing of Pol II near the transcription start site [21], in which a higher binding ability indicates the increased pausing of RNA Pol II within the core promoter. Surprisingly, by analyzing the enrichment of the MTSS1 core promoter or a distal promoter region (Figure 5E, upper) after RNA-Pol-II-ChIP experiments, we observed that the binding potential of Pol II to the MTSS1 core promoter was largely increased in SNHG15-expressing cells compared to the cells without SNHG15 expression (Figure 5E, lower left). No enrichment of the distal promoter region was seen (Figure 5E, lower right). These results, in combination with the data in Figure 2B, strongly suggest that the SNHG15-mediated transcriptional repression of MTSS1 is via stalling or arresting RNA Pol II at the core promoter.

### 2.6. Interaction of SNHG15 with the MTSS1 Core Promoter Mediates MTSS1 Transcription

**Figure 6:**
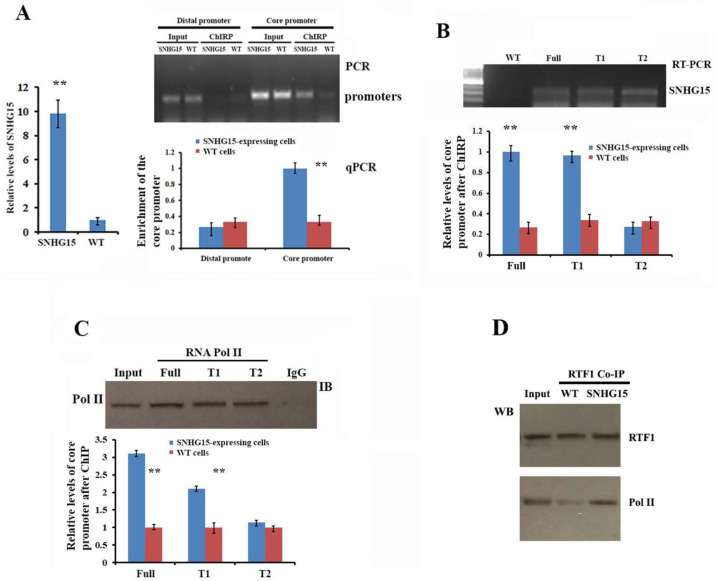
Interaction of SNHG15 with the MTSS1 core promoter repressed MTSS1 mRNA expression. (**A**) ChIRP assays were performed to detect potential interaction of SNHG15 with the MTSS1 promoter. Two set of primers as shown on Figure 5E were used to amplify the distal or core region of the MTSS1 promoter. Left: RT-qPCR showed the enrichment of SNHG15 after ChIRP assays. Right upper: PCR and agarose gel electrophoresis indicated that SNHG15 favorably interacted with the MTSS1 core promoter. Right lower: RT-qPCR confirmed that SNHG15 effectively bound to the MTSS1 core promoter. ** *p* < 0.01. (**B**) Upper: RT-PCR and agarose gel electrophoresis displayed full and truncated SNHG15 instances that were precipitated in ChIRP experiments. Lower: qPCR determined that the 5′ region (T1), but not the 3′ region (T2), of SNHG15 bound to the MTSS1 core promoter. ** *p* < 0.01. (**C**) ChIP experiments using RNA Pol II antibodies were performed in breast cancer cells expressing full-length or truncated SNHG15. Upper penal: Western blots indicated precipitated Pol II after ChIP assays. Lower: qPCR after Pol II-ChIP showed higher enrichment of the core promoter in the cells expressing full-length SNHG15 while the enrichment was notably decreased in cells expressing T1 truncate. No enrichment was observed in cell expression T2 that did not interact with the core promoter. ** *p* < 0.01. (**D**) Co-IP and Western blots were performed using RTF1 antibody to detect the potential interaction between RTF1 and Pol II in cells expressing SNHG15.

We hypothesized that the interaction of SNHG15 with the MTSS1 promoter is required to repress MTSS1 transcription. To address this, we performed ChIRP (chromatin isolation by RNA precipitation) assays to investigate whether SNHG15 directly binds to the MTSS1 core promoter [22]. Two sets of primers, one set that amplified a distal region of the MTSS1 promoter and another set that amplified the core promoter region, were utilized for qPCR. ChIRP followed by qPCR experiments revealed that SNHG15 was favorably associated with the MTSS1 core promoter (Figure 6A). Furthermore, ChIRP assays in stable cell lines expressing full-length or truncated SNHG15 showed that the full-length and 5′ region (T1 truncate), but not the 3′ region (T2 truncate), interacted with the MTSS1 core promoter (Figure 6B). We then used ChIP assays to analyze the effects of individual SNHG15 truncates on the binding potential of Pol II to the MTSS1 core promoter. Consistently, cells expressing full-length SNHG15 demonstrated a highest binding occupancy of Pol II to the MTSS1 core promoter. No binding occupancy changed in the cells expressing the T2 truncate because it did not interact with the MTSS1 core promoter. However, although the T1 SNHG15 truncate interacted with the core promoter, it showed a relatively lower binding occupancy than the full-length of SNHG15; apparently, it lacked the 3′ region that binds to RTF1 (Figure 6C). Finally, Co-IP experiments detected that SNHG15 increased the binding potential of RTF1 to RNA Pol II (Figure 6D). These data suggested that the SNHG15-mediated transcription repression of MTSS1 mRNA was contributed to by two ways. One way was through the interaction of the 5′ region of SNHG15 with the MTSS1 core promoter. The second was through the 3′ region of SNHG15 that carried and delivered RTF1 to contact RNA Pol II at the transcription start site. Both ways affected the activity of the core promoter via stalling or arresting transcriptional initiation or elongation.

### 2.7. A Model of SNHG15-Mediated Suppression of MTSS1 Gene Transcription

**Figure 7:**
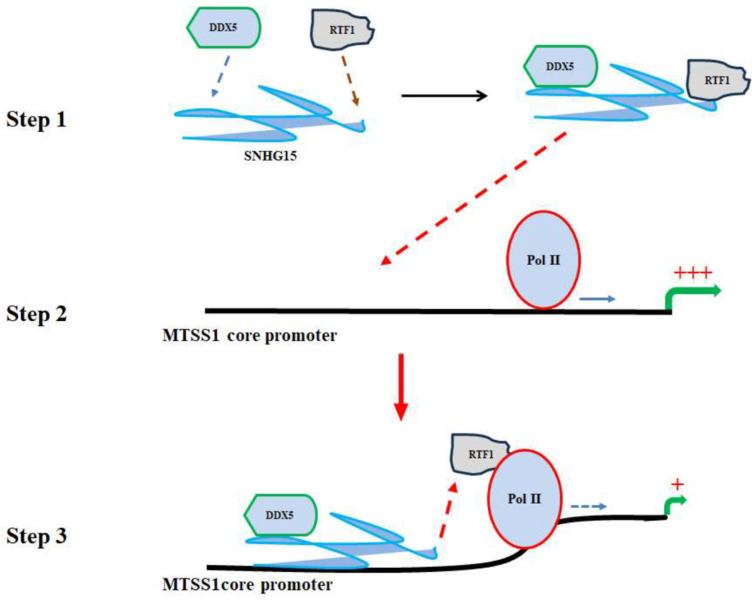
A model of SNHG15-mediated repression of MTSS1 transcription. Step 1: In the nucleus, SNHG15 complexes with DDX5 and RTF1. The middle part of SNHG15 associates with DDX5 and its 3′ region binds to RTF1. Step 2: Subsequently, the 5′ region of SHNG15 interacts with the core promoter of the MTSS1 gene. This interaction not only changes the architecture of the DNA promoter but also allows SNHG15 to transport its 3′-associated RTF1 to the core promoter and contact with RNA Pol II. Step 3: Binding of SNHG15 to the core promoter interferes with MTSS1 transcription while interaction of RTF1 with RNA Pol II regulates the pausing process, thus resulting in repression of MTSS1 transcription.

Based on the investigation, we predict a model by which SNHG15 mediates MTSS1 transcription (Figure 7). First, the nuclear localized SNHG15 forms a complex with DDX5 and RTF1. Second, the 5′ region of SNHG15 interacts with the core promoter of the MTSS1 gene; this interaction may change the local DNA architecture and interfere with RNA-polymerase-II-directed-transcription initiation. Third, interaction with the core promoter allows SNHG15 to deliver its associated RTF1 to the RNA Pol II complex near the core promoter, thus enhancing the stalling of Pol II at the core promoter. SNHG15 truncates with a diminished ability to interact with the MTSS1 promoter or lacking the ability to deliver RTF1 to the core promoter are unable to repress the transcription of the MTSS1 gene.

## 3. Discussion

The central dogma of gene expression states that DNA is transcribed into messenger RNAs that serve as the template for synthesizing proteins. The discovery of a large numbers of long RNA transcripts that do not code for proteins (lncRNAs) provides an important insight on the centrality of RNA in gene regulation. An emergent theme is that lncRNAs form extensive networks through interaction with DNA, RNA, and proteins to modulate the chromatin structure and mediate the transcription of target genes.

SNHG15, an lncRNA with the size of about 983 bp, has been shown to be involved in many human malignancies [16,17]. SNHG15 expression is positively associated with larger tumor sizes and the lymph-node metastasis of breast tumors. In this study, we identified a novel function of SHNG15 to transcriptionally mediate MTSS1 gene expression. Our study not only demonstrated that SNHG15-mediated MTSS1 suppression promotes breast cancer progression but also revealed a molecular mechanism by which an lncRNA like SNHG15 could serve as a regulator to repress gene transcription via interacting with the gene promoter.

MTSS1 is a tumor-suppressive gene that functions in the inhibition of metastasis via regulating actin dynamics [23]. The MTSS1 gene could be epigenetically regulated since previously, studies had shown that the methylation of the MTSS1 promoter in bladder cancer cell lines correlated with MTSS1 silence and that increased MTSS1 promoter methylation in gastric cancer cell lines and tissue samples reduced endogenous MTSS1 expression [24]. Our study provided molecular evidence that MTSS1 down-regulation was mediated by SNHG15 and that this mediation was apparently through inhibiting the MTSS1 promoter activity (Figure 5). The preliminarily investigation on human tumors further showed that the negative connection between SNHG15 and MTSS1 mRNA might also occur in human breast tumor tissues. Since the sample size in our study was relatively small, future studies are necessary to collect and use large population sizes to confirm the corelative expression of SNHG15 and MTSS1 mRNA in human breast tumors.

One of the biological functions of lncRNA is as a regulatory molecular to modulate gene expression [25]. lncRNAs can interfere with transcription by interacting directly with the target gene promoter to repress or activate the gene. For example, in studies, the lncRNA Airn caused the transcriptional pausing of Igf2r mRNA at the gene promoter [26] and GNG12-AS1 affected the transcriptional initiation of DIRAS3 mRNA [27]. Our study determined that MTSS1 suppression was dependent on the interaction between the 5′ region of SHNG15 and the MTSS1 core promoter. This interaction resulted in two consequences. One was to interfere with the transcriptional initiation through changing the local promoter architecture. The other was to deliver RTF1 to associate with RNA Pol II at the transcription start site to cause transcription pausing. Both roles increased the occupancies of RNA Pol II on the core promoter and halted the transcriptional initiation or elongation of the MTSS1 gene. In addition, to exert its function, SNHG15 required two RNA-binding proteins: DDX5 and RTF1. Interaction with DDX5 increased SHNG15 stability while binding to RTF1 allowed SNHG15 to be a transporter to deliver the protein to RNA Pol II at the core promoter, thus enhancing MTSS1 suppression.

In conclusion, our study revealed a novel mechanism whereby SNHG15 can serve as a transcriptional regulator to silence MTSS1 mRNA expression through interaction with the gene core promoter and causing RNA Pol II pausing.

## 4. Materials and Methods

### 4.1. Cell Lines and Culture Conditions

Human breast cancer cell lines MDA-MB-231, BT-549, and HEK293T were obtained from the American Type Culture Collection (ATCC). Breast stable cell lines expressing MS2-tagged full-length and truncated SNHG15 were established as previously described [19]. Cells were cultured in Dulbecco’s modified Eagle’s medium (DMEM) supplemented with 10% fetal bovine serum (FBS), 100 U/mL penicillin, and 100 µg/mL streptomycin at 37 °C in a humid environment with 5% CO_2_.

### 4.2. Reagents

Primary antibodies against human RNA Pol II, MTSS1, and RTF1 were purchased from Cell Signaling Co. (Danvers, MA, USA). DDX5 and GPDH antibodies were purchased from Boster Biological Technology Co. (Wuhan, China). Secondary antibodies conjugated with horseradish peroxidase (HRP) were purchased from Santa Cruz Biotechnology (Dallas, TX, USA). siRNAs against SNGH15, MTSS1, DDX2, and RTF1 mRNAs were purchased from Gene Pharma (Suzhou, China). PCR primers used in the study were obtained from IGE Biotechnology (Guangzhou, China) as listed in Supplementary Table S1.

### 4.3. Cell Proliferation Assay

Cell proliferation assays were performed as previously described [19]. Briefly, freshly cultured cells were seeded in 96-well plates at a density of 5 × 10^3^ cells per well in a 100 μL volume. Cell proliferation was determined using a MTT kit (Sigma, Livonia, MI, USA) for a period of 2, 24, 48 or 72 h. Each experiment was repeated three time with four replicates.

### 4.4. Transwell Assay

Invasion assays were performed using 24-well transwell pre-coated with Matrigel (BD Biosciences, Franklin Lakes, NJ, USA). Fresh cells were cultured at 37 °C for 24 h. A total of 2 × 10^4^ cells in 200 μL serum-free medium were added to the upper layer of the insert, which was prewarmed at 37 °C for 2 h. To lower layer, we added 500 μL DMEM medium with 10% FBS as the chemoattractant. Cells were counted after 24–36 h incubation.

### 4.5. Cell Extract Preparation

Total cell lysates and extracts of nuclear and cytosolic fractions of BT549 and MDA-MB-231 cells were prepared as previously described [28]. Briefly, cells were harvested and washed with cold PBS. Four volumes of ice-cold cytosolic buffer (10 mM HEPES, pH 7.6), 1X protease inhibitor cocktail (Roche diagnostics), 60 mM KCl, 1 mM EDTA, and 0.075% (*v*/*v*) NP40) were added to the cells. After incubation on ice for 20 min, the cytosolic fraction was collected by centrifugation at 1000× *g* for 5 min at 4 °C. Supernatant was collected in the form of cytoplasmic extracts. Undissolved pellet was washed twice with cold PBS and resuspended in 2 volumes of buffer containing 0.45 M NaCl in 10 mM Hepes (pH 7.4) on ice for 15 min. After centrifugation at 18,000× *g* for 5 min at 4 °C, the resulting supernatant was collected in the form of the nuclear extracts.

### 4.6. MS2-Tagged RNA Pulldown Experiments

RNA-pulldown assays were performed as previously described [19]. Briefly, cells expressing MS2-tagged full-length or truncated SNHG15 were cultured and lysed. Lysates were incubated with a recombinant MBP-MCP at 4 °C overnight and then incubated with maltose beads (NEB, Ipswich, MA, USA) overnight with gentle rotation. After extensive washing with cold PBS, SNHG15 and its associated proteins were eluted. Enrichment of precipitated SNHG15 was verified by qRT-PCR and proteins co-precipitated with SNHG15 were identified by Western blots.

### 4.7. RIP (RNA Immunoprecipitation)

RNA immunoprecipitation was performed as previously described [28]. Briefly, cells were lysed in an ice-cold lysis buffer containing protease and RNase inhibitor. After high-speed centrifugation for 60 min at 4 °C, supernatants were obtained and incubated with DDX5 or RTF1 antibodies for 2 h with gentle rotation. Protein A agarose beads (Sigma, Livonia, MI, USA) were added to the solution and continuously incubated at 4 °C overnight in the presence of RNase inhibitor (250 units/mL). The supernatant was removed and the beads were extensively washed in lysis buffer. After proteins were eluted from the beads, half elution material was used to analyzing immunoprecipitated protein and the other part was used to analyzing associated RNA by qRT-PCR.

### 4.8. Western Blots

Cells were freshly cultured overnight and lysed in the presence of protease and nuclease inhibitors. Protein concentration was determined using a BCA Protein Assay Kit (Shanghai, China). Equal amounts of proteins were electrophoresed in 4–12% SDS-PAGE and transferred onto 0.45 μm nitrocellulose membranes. The membranes were probed with primary antibodies (1:500 to 1:1000) overnight at 4 °C with gentle rotation. After extensively wash, blots were incubated with respective HRP-conjugated secondary antibodies (1:2000 to 1:5000) for 1 h. The blots were incubated with chemiluminescence reagents (Thermo Scientific, Waltham, MA, USA). For sequential blotting experiments, the membranes were washed with Blot Stripping Buffer (Thermo Scientific, Waltham, MA, USA) for 1 h and re-blocked with fat-free milk. Membranes were incubated with other primary antibodies if necessary.

### 4.9. Specimens

A total of 33 human breast tissue samples, which included 24 breast tumors and 9 paracancerous mammary tissues, were collected from patients who had undergone surgery at the Shantou University Tumor Hospital (Shantou City, Guangdong Province, China) in 2023. All human breast samples were received as discarded tissue (excess tumor tissue after enough specimens were collected by the Shantou Tumour Hospital, Pathology Department for diagnostic tests) and did not contain a code derived from individual personal information. The experiments were approved by the Shantou Tumour Hospital and operated according to International Conference on Harmonisation (ICH)/WHO GCP and the applicable laws and regulations.

### 4.10. Total RNA Extraction from Breast Tissue Specimens and Reverse Transcription

Breast tissue specimens (~20 mg) were homogenized in 1 mL RLT reagent (Qiagen, San Diego, CA, USA) using a high-speed homogenizer (16,000 rpm). Total RNA was extracted from malignant (n = 24), and paracnacerous mammary tissues (n = 9) using a RNeasy Mini Kit (QIAGEN, San Diego, CA, USA). RNA concentration was quantified using a Nanodrop ND-2000c Spectrophotometer. Prepared RNA was used immediately or stored at −80 °C. For cDNA synthesis, 1 μg of total RNA was reverse-transcribed using a kit from Beyotime Biotech (Shanghai, China).

### 4.11. Quantitative Real-Time PCR

qRT-PCR was carried out in a Fast PCR system (ABI 7500, Applied Biosystems, Waltham, MA, USA) using SYBR Green PCR Master Mix (Qiagen, San Diego, CA, USA) according to the manufacturer’s instructions. For quantification, values of target mRNAs were normalized to internal GAPDH mRNA control. Relative expression was calculated using the 2^−∆∆Ct^ method (Ct, cycle threshold). All primers used in qPCR are listed in Supplementary Table.

### 4.12. Luciferase Assays

PsiCHECK 2 was purchased from Promega (Madison, WI, USA) and used for luciferase reporter construction and luciferase assays. To construct the luciferase reporters, different lengths of the MTSS1 promoter were PCR-amplified and cloned into the 5′ end of the Renilla luciferase gene of the PsiCHECK 2 plasmid. Primer pairs used in the cloning experiments are listed in Supplementary Table. All constructs were verified by sequencing analysis. BT-549 or MEK293T cells were transfected with individual psiCHECK-2 constructs containing different region of the MTSS1 promoter using Lipofectamine 3000 Reagent (Invitrogen, Waltham, MA, USA) according to the manufacturer’s instructions. After transfection for 36 h, the cells were harvested for Firefly/Renilla luciferase assays using the Dual-Luciferase Reporter Assay System (Promega, Madison, WI, USA). Luciferase activities were normalized to the empty psiCHECK-2 plasmid.

### 4.13. Chromatin Immunoprecipitation (ChIP)

Cells overexpressing SNHG15 or truncated SNHG15 variants were grown to more than 80% confluence in 10 or 15 cm culture plates. ChIP assays were performed using a kit from Beyotime Biotech according to the manufacture’s instruction. Briefly, cultured cells were crosslinked with 1% formaldehyde for 10 min followed by adding to quench residual formaldehyde and washed three times with cold PBS. Cells were lysed and centrifuged in a microcentrifuge at 1000 rpm for 5 min in 4 °C. After removing supernatants, pellets were resuspended in 0.5 mL of lysis buffer and sonicated to break down chromatin into appropriate small fragments. Nuclear debris was removed by centrifugation and the supernatant was aliquoted and stored at a −80 °C freezer. For ChIP assays, anti-RNA Pol II antibodies or normal IgG samples were mixed with nuclear extract and incubated at 4 °C overnight with gentle rotation. The extracts were then incubated with Protein A beads for 4 h at 4 °C to capture DNA fragments associated with the protein. The beads were extensively washed with buffers and the proteins were eluted and reverse cross-linked to free the proteins. Aliquots of solution were used for analyzing precipitated proteins by Western blotting. The rest was used for DNA isolation and qPCR. Agarose gel electrophoresis was carried out to confirm the PCR products. Primers used in PCR or qPCR are listed in Supplementary Table S1.

### 4.14. Chromatin Isolation by RNA Purification (ChIRP)

The ChIRP experiments were performed essentially using a protocol as previously described [10]. Briefly, confluent BT-549 or MDA-MB-231 cells expressing MS2-tagged full-length SNHG15 or SNHG15 truncates were washed in plates with PBS, then treated with 1% formaldehyde for 10 min. Formaldehyde was quenched with 125 mM glycine for 5 min. Cells were then scrubbed from the plates and centrifuged for 10 min at 3000 rpm. After aspirating all liquid, cells were resuspended in lysis buffer and sonicated for 5 min until lysate was clear. Sonicated cell lysates were centrifuged at 15,000 rpm for 10 min at 4 °C and the supernatant was transferred to fresh tubes. To pull down MS2-tagged SNHG15, amylose resin conjugated with recombinant MBP-MCP was incubated with the supernatant at 4 °C overnight in the presence of RNase and protease inhibitors with gentle rotation. After extensive washing, SNHG15-MS2 was eluted with 100 μL lysis buffer containing 20 mM maltose. Aliquots of the eluted material were used to extract RNA for analyzing enrichment of SNHG15 by qRT-PCR. The remaining material was used for analyzing potential DNAs associated with SNHG15 by qPCR.

### 4.15. Statistical Analysis

Statistical significance was measured using Student’s *t*-test (two-tailed). A *p* < 0.05 was considered significant. Statistical differences between more than two groups were evaluated by one-way ANOVA followed by Tukey’s multiple comparison test. All numerical values described represent means ± SD.

## Data Availability

All data generated or analyzed during this study have been included in this published article and its Supplementary Information files.

## Supplementary Materials

The following supporting information can be downloaded at: https://www.mdpi.com/article/10.3390/ijms252111565/s1.
