# Supplementary material for: SNHG15 Mediates MTSS1 Gene Expression via Interacting with the Gene Promoter and Regulating Transcription Pausing
Source: Int J Mol Sci. 2024 Oct 28;25(21):11565. doi: 10.3390/ijms252111565 (PMC11546481; doi:10.3390/ijms252111565)
Supplement: Supplementary file 1 [file ijms-25-11565-s001.zip › ijms-3196362-supplementary.pdf]

Suppl Figure S1

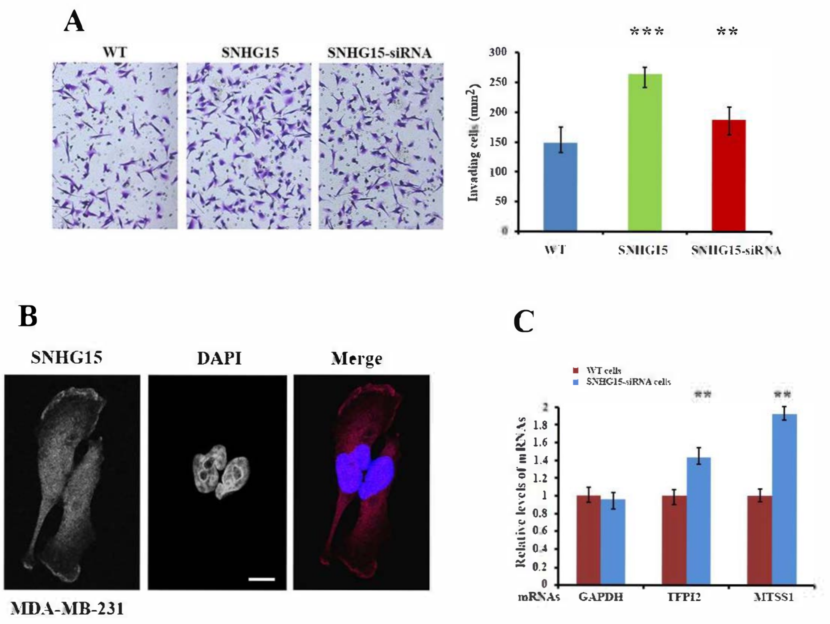

Suppl Figure S2

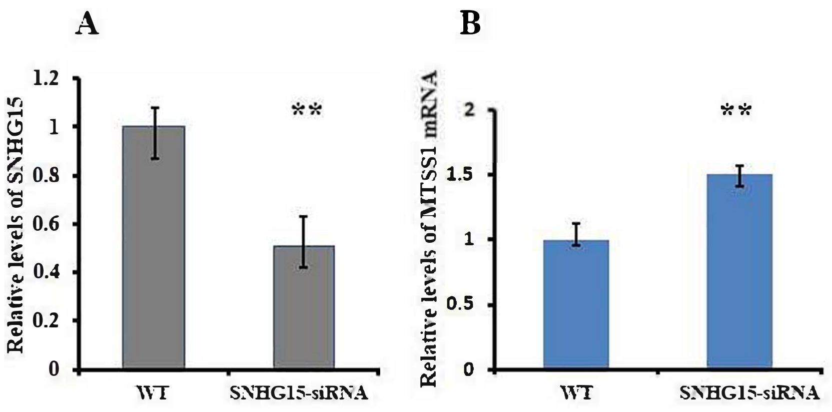

MDA-MB-231

Suppl Figure S3

A

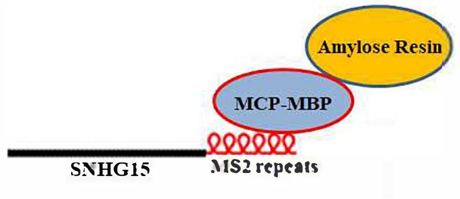

B

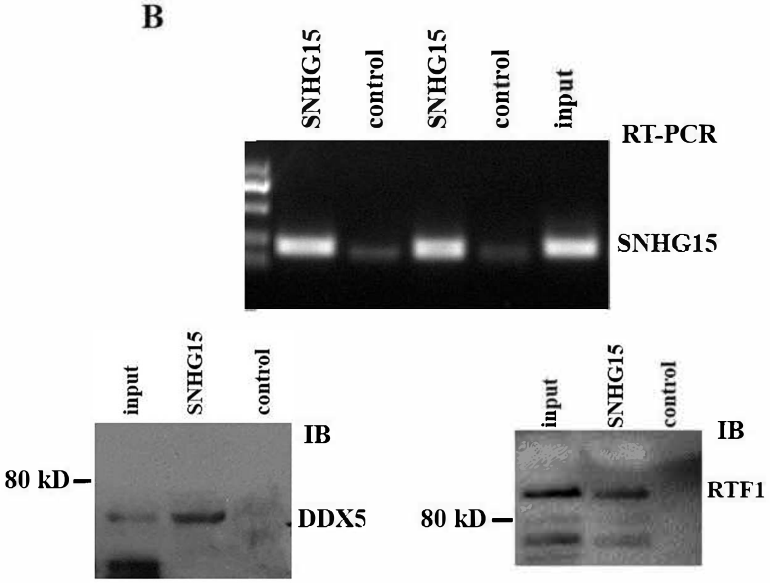

Suppl Figure S4

```
-500
 TTAATTATATATTTCGCTATTTGCAATCAGGACTCGGCGATGCAGTCCTGACATCAGAGA
 ATGGGCGTCTTCTCAGAAGGCCGCAGTAGCGGCAGCAGCTTTCCCGCCCTACTGAGCGCT
 CACTACAAGCGGGCTCTGGGCTAGGCGCGCCACCCGTGCAAGTCCCCGGGGAGCGGCGGT
 GCACCCTCCGTCCCGCGCGCTCGCAGCCATTGTAGGGGTGGGCGCTCGCCAGGCAGGGTG
 CCGACACGCCCTCTCCGCGCTGCGACGGGCGGCCGGGGGAGGAGAGGGTGCGCTGTGCGC
 ACCGGAGGGAGAGGCTCCGGCCCAGCGCCGCCTGCCCGCCAGCAGACCAGCAGGCTCCTC
 TTTGCGGCGGGTGCCCCACGCGCCTCTCGGGCGCGCGCTCCCAGCCCCGCCCGCGTGCGG
 CGGCCGCACGGCCGCGAGAGCGCGCCTCCGCTTGGCGGGCACGGCCGCGAGCGCGCGTGC
 GCCCCTCCGAACGTCCCCGG
                    -1
```

The nucleotide sequence of the MTSS1 core promoter is shown
Red sequences indicate potential binding site for RNA Pol II.

Supplemental Table S1, Primers, siRNAs and FISH probes used in the study

**PCR Primers**	**Sequences**
GAPDH Forward	GAGTCAACGGATTTGGTCGT
GAPDH Reverse	TGGGATTTCCATTGATGACA
SNHG15 Forward	GGGACTACGCGGTGACGTCG
SNHG15 Reverse	CACATATTTAAATCCATATTTATTAA
SNHG15 truncate-1 F	GGGACTACGCGGTGACGTCG
SNHG15 truncate-1 R	TCGCCTCCCAGTTTCATGGAC
SNHG15 truncate-2 F	GTCCATGAAACTGGGAGGCGA
SNHG15 truncate-2 R	TCACATATTTAAATCCATATTTATTAA
SNHG15 truncate-3 F	TAAATCACGTTGCAGAGACCCCA
SNHG15 truncate-3 R	ACACAGGCAGGCCTCATCCCTG
MTSS1 Forward	ACATGGCCACCAACACACGTG
MTSS1 Reverse	CGAAAACTGCCTCAGCTTGGCT
TFPI-2 Forward	GTCCCAAGAAGACAAAGTCGCA
TFPI-2 Reverse	GTGGTCTCCAACCCACAATGTC
TIMP3 Forward	TTCCTCCTTTGGGCATCT
TIMP3 Reverse	ACACTTGGGTGCCATCTT
MYCT1 Forward	GCCAGAAAACTTTTGGGAGGA
MYCT1 Reverse	ATCCAGTTCTGTTGAGGCCG
MTSS1 core promoter For	AGCATCCTGCAACTTTATCGAAC
MTSS1 core promoter Rev	CTTGTAGTGAGCGCTCAGTAGG
MTSS1 distal promoter For	AACCAGGGTTGGATTCCTTCCAAC
MTSS1 distal promoter Rev	CCTTTCTGGGCCTCAGTTTCCTC
**MTSS1 promoter subcloning primers**	
MTSS1 promoter p2000 For	aattaGAGCTCGGGAAAAAGGCCACAAGAGAGG
MTSS1 promoter p2000 Rev	aattaGCTAGCGACGTTCGGAGGGGCGCACG
MTSS1 promoter p1470 For	aattaAGGCCTTACAGATGGATGAGGAAACTGAG
MTSS1 promoter p1000 For	aattaAGGCCTTCCCACTCCCCCCAGTCTTTTC
MTSS1 promoter p550 For	aattaAGGCCTGGATACGCCCCCGAACTCTCC
MTSS1 promoter p1500 Rev	aattaGCTAGCCCTTTCTGGGCCTCAGTTTCCTC
**siRNAs**	**Sequences**
For MTSS1 mRNA	CGGCCAGTGATTGAAGAAGAA
For RTF1 mRNA	AATGGCGTGCTGCCTTCTGT
For DDX5 mRNA	CAGUAAAGUUUUCGGGUUA
For SNHG15	UAGGUGUAUUAACACAGGCTT

FISH probes	Sequences
For SNHG15 -1	gtctctgcaacgtgatttac
For SNHG15 -2	ggttcagagaaaacgcaggc

Supplemental figures

Supplemental Figure S1

(A) Representative images (left) of Transwell assays showed that increased expression of SNHG15 in MDA-MB-231 cells enhanced cell invasive ability (right). ** $p < 0.01$, *** $p < 0.001$. (B) FISH was performed to determine the subcellular localization of SNHG15 in MDA-MB-231. Substantial amount of SNHG15 was localized in the nucleus. Scale bar: 10 µm. (C) Two transcripts, MTSS1 and TFPI2 mRNAs, and a control GAPDH mRNA were selected to confirm their expression in MDA-MB-231 cells. qRT-PCR showed that levels of both mRNAs were reduced after knocking down SNHG15 expression by siRNA.

Supplemental Figure S2

(A) SNHG15 levels was examined by RT-qPCR in MDA-MB-231 cells after siRNA treatment. (B) qRT-PCR indicates that MTSS1 mRNA was significantly reduced in responding to downregulation of SNHG15. **$P < 0.01$.

Supplemental Figure S3

(A) Schematic drawing showing that SNHG15-MS2 chimeric RNA can be specifically complexed with MBP-MCP, and bound to amylose resin. (B) Upper: SNHG15-MS2 was pulled down by MBP-MCP conjugated to amylose resin. Co-precipitated DDX5 and RTF1 with SNHG15 were shown by western blots.

Supplemental Figure S4

Nucleotide sequences of the MTSS1 core promoter

Supplemental Table S1

PCR primers, siRNA and FISH probes used in this study
